# Dose dependence of *Phasmarhabditis* isolates (*P*. *hermaphrodita*, *P*. *californica*, *P*. *papillosa*) on the mortality of adult invasive white garden snails (*Theba pisana*)

**DOI:** 10.1371/journal.pone.0270185

**Published:** 2022-07-22

**Authors:** Jacob Schurkman, Irma Tandingan De Ley, Adler R. Dillman

**Affiliations:** Department of Nematology, University of California, Riverside, California, United States of America; University of Limpopo, SOUTH AFRICA

## Abstract

*Theba pisana* is an invasive snail pest which has established itself in San Diego County and some areas of Los Angeles County, California. The snail has grown to large populations in some areas and mitigation is becoming necessary to stop the spread of the species. In a previous study, three US strains of *Phasmarhabditis* species (*P*. *californica*, *P*. *papillosa*, and *P*. *hermaphrodita*) effectively killed juvenile (0.25 gram each, 4–6 mm wide) *T*. *pisana* in laboratory conditions at 5 times (150 IJs/cm^2^) the recommended dose. Based on laboratory assays, we demonstrated that the same three US strains of *Phasmarhabditis* can effectively kill larger adult *T*. *pisana* (0.4–1.2 gram, 11.5-15mm wide) in two weeks at the same dose. The strains were more efficient at killing *T*. *pisana* than the compared molluscicide Sluggo Plus®. Results further showed that the most virulent *P*. *californica* did not effectively kill *T*. *pisana* at lower doses of 30 IJs/cm^2^ and 90 IJs/cm^2^. Additional research is needed to develop the most efficient means of application of *Phasmarhabditis* to mitigate *T*. *pisana* in the field.

## Introduction

Terrestrial snails and slugs belong to the class Gastropoda (Phylum: Mollusca). They play important roles throughout a variety of ecosystems where they act as detritivores and plant feeders, inherently breaking down plant materials and fertilizing the soils they occupy [1,2]. While many terrestrial gastropods are thought of as pestiferous nuisances which invade agricultural spaces and damage produce, multiple native species only occupy specific niches where they serve critical roles as detritivores such as banana slugs *Ariolimax* Mörch 1859 or other slugs [3]. However, many terrestrial gastropods are invasive agricultural pests, threatening native biodiversity [4–7]. In California, it is estimated that there are about 279 species of terrestrial gastropods, however 37 of those species are invasive [8]. These invasive species are the gastropod pests which are typically found throughout the agricultural industry [8]. They are hypothesized to have arrived in California via horticultural trade when infested produce products were delivered for trade [9,10]. Some of these invasive gastropod species can cause agricultural damage where crop yields are significantly reduced upon introduction of the pests [11–13].

Gastropods are also capable of spreading plant and human diseases. Terrestrial gastropods have been found to harbor *Alternaria brassicicola*, the causative agent of black leaf spot, and other pathogenic fungi [14–16]. They are also thought to be partially responsible for salad crop recalls after both *Campylobacter spp*. and *Escherechia coli* were reported in the feces of some gastropods [17,18]. Multiple gastropod species have also been found to harbor the human parasite *Angiostrongylus cantonensis*, the causative agent for rat lung worm disease [19].

**Fig 1:**
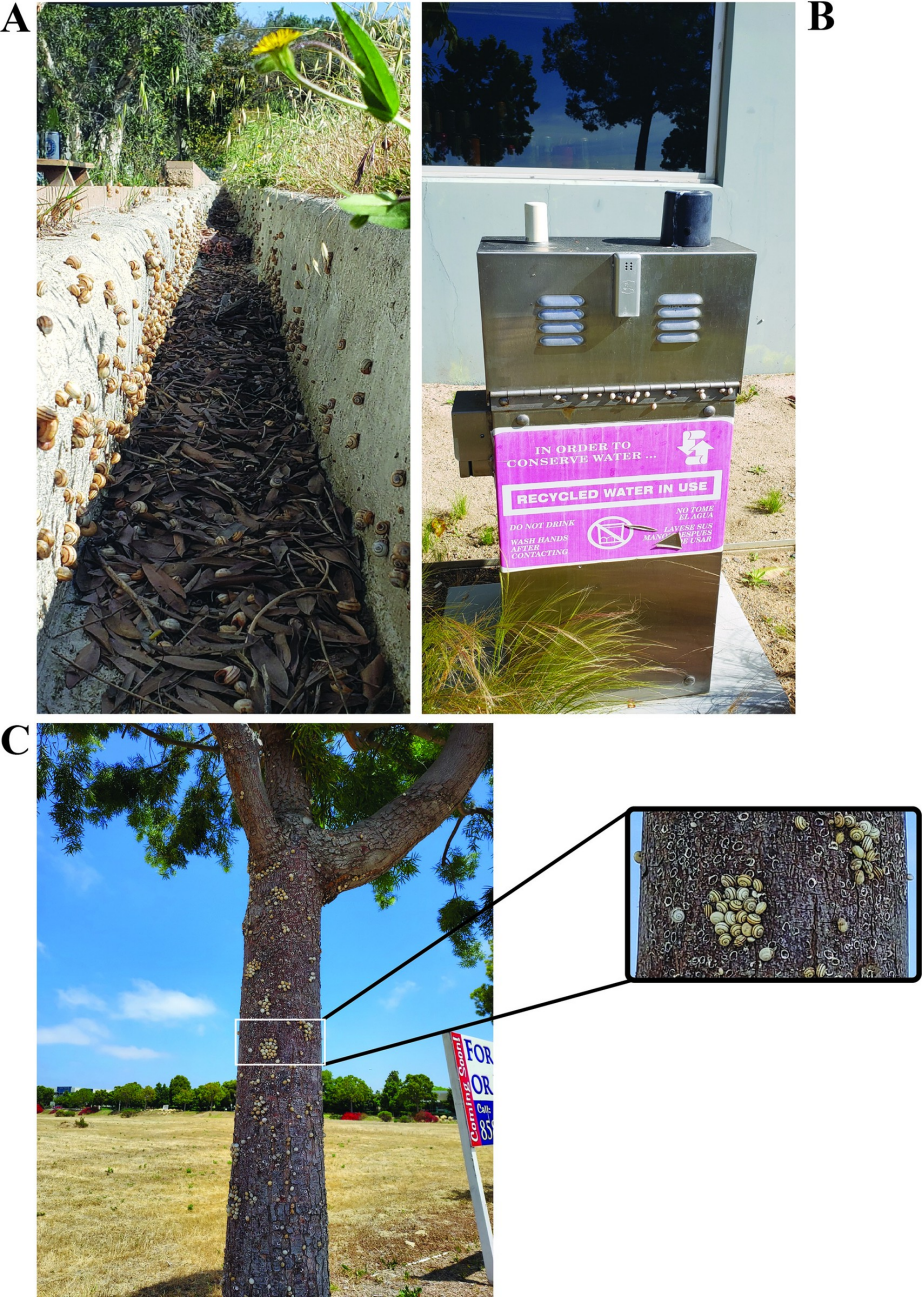
A gutter cluttered with *Theba pisana* behind a local business in Oceanside, CA (A). A utility box covered with aestevating *T*. *pisana* beside a local business in Oceanside, CA (B). Clusters of aestivating *T*. *pisana* on a tree beside a sidewalk in Oceanside, CA (C).

*Theba pisana* (Müller 1774) is an invasive gastropod species also known as the Italian white snail. It has been introduced to various countries across the globe [20]. The snails have become an important pest throughout most of the countries they have invaded, where extremely dense populations occur [21,22]. They are known to be active during the wet periods of the year when they feed on the leaves and stems of plants. During the hot and dry periods, they aggregate in large clusters where they crawl up large stalks and aestivate [21,22]. They can cause damage to a variety of different plants like ornamental flowers, vegetables, citrus, almond and olive trees, and grapevines [21,23–25]. They have also been found to cause damage to farming machinery by clogging equipment which takes up large clusters of snails on plant material and to livestock by causing livestock to reject hay which is heavily infested with *T*. *pisana* [22,23,26,27]. In California, *T*. *pisana* is considered a B-rated pest and is mostly present in southern sections of California near the location it was first isolated in La Jolla, San Diego CA [28]. A B-rated pest is simply a pest with limited distribution that is known to cause economic or environmental detriment in California. Rating the pest allows the state to take specific governmental actions against the pest upon finding it within the state. *T*. *pisana* has been noted in Los Angeles County, and was recently reported in Half Moon Bay, San Mateo County, CA (https://agwm.smcgov.org/white-garden-snail). The snails cause major aesthetic disturbances on public and private properties and sometimes render facilities unusable (Fig 1) [29]. Although the snails are currently restricted to certain areas of distribution, this displays significant potential to damage natural ecosystems or agriculture, human health, or commerce; and is suggested to be of top national quarantine importance in the US [30].

Mitigation of the Italian white snail in California is necessary to protect local biodiversity and public health. Methods to control populations of *T*. *pisana* have included metaldehyde baits, sprayable molluscicides, burning, barricading/trapping, and hand picking [31–34]. While some of these methods have proven effective [35,36], they are not targeted methods of pest control and may be toxic to native mollusks (32) and a variety of other organisms [37]. Molluscicide use also provides the possibility of developed resistance over a prolonged period of exposure [38]. It has been discovered that some snails have already developed resistance to metaldehyde baits, one of the more commonly used methods of gastropod pest control [39]. They can also contaminate groundwater as they leach into the soil [40].

The use of biological control agents to manage gastropod pests is another method of gastropod control that is considered safer and more environmentally sustainable. Biological control can be more targeted methods of integrated pest management (IPM). *Phasmarhabditis hermaphrodita* (Schneider 1859) is the most well-known example of a successful biological control agent against pestiferous gastropods. The nematode was discovered in Europe where it has seen extensive commercial and home use under the brand Nemaslug® (BASF Agricultural Solutions, United Kingdom). *P*. *hermaphrodita* is an effective biological control agent in a variety of different environments including fruit, vegetable, and ornamental crops across many European locations [29,41–44]. It is a parasitic nematode that infects gastropods. *P*. *hermaphrodita* was found safe to various species of earthworms, as well as native, non-pest European slugs and snails, though additional non-target testing is needed [45–48].

Recently, three *Phasmarhabditis* species were discovered in California including *P*. *californica*, *P*. *hermaphrodita*, and *P*. *papillosa* [49–51]. Pathogenicity assays have been performed using each of these local strains and it was found that they caused significant mortality in *Deroceras reticulatum* in laboratory and field simulated conditions [44,52]. The US strains have also been tested against small (4-6mm) *T*. *pisana* in controlled lab conditions where all strains caused 100% mortality within 5 days. The strains’ efficacies were compared to the molluscicide Sluggo Plus® (Monterey Lawn and Garden, Fresno CA, USA) and the nematodes were shown to be equally as effective at causing mortality in *T*. *pisana*. However, larger *T*. *pisana* exist in large populations throughout the seasons of the year [53] (Fig 1). *T*. *pisana* are generally considered adults once their shell size surpasses 10mm [54]. Their maturity can also be assessed based on the time of year and size differences within a population [55]. In the past, larger gastropods were found to be more resistant to *Phasmarhabditis* [56,57]. Therefore, in order to assess the efficacy of the US *Phasmarhabditis* strains against *T*. *pisana*, their lethality should be determined when applied to both large and small snails.

Another important aspect of evaluating the efficacy of a biological control agent is the minimum effective dose. The recommended dose when applying Nemaslug® is 30 infective juveniles (IJs)/cm^2^. When US *Phasmarhabditis* strains were originally tested against *T*. *pisana*, a 5-fold dose of 150 IJs/cm^2^ was used [29]. This was done only to show that the US strains were capable of killing the snails. A lower dose of nematodes is more feasible and preferable in both economic and production terms. A decreased and more economically sound and effective dosage of US *Phasmarhabditis* species should be determined against *T*. *pisana*.

We tested the efficacy of three US strains of *P*. *californica* (ITD726), *P*. *hermaphrodita* (ITD272), and *P*. *papillosa* (ITD510) and Sluggo Plus® against larger adult *T*. *pisana* (11.5-15mm). Based on the comparative efficacy of these three species, we further tested *P*. *californica* at lower doses of 30 IJs/cm^2^ (Nemaslug ®-recommended dose) and 90 IJs/cm^2^ against the larger-sized and heavier *T*. *pisana*.

## Methods

### Size dependence assay on *Theba pisana* exposed to Phasmarhabditis *spp*.

**Fig 2:**
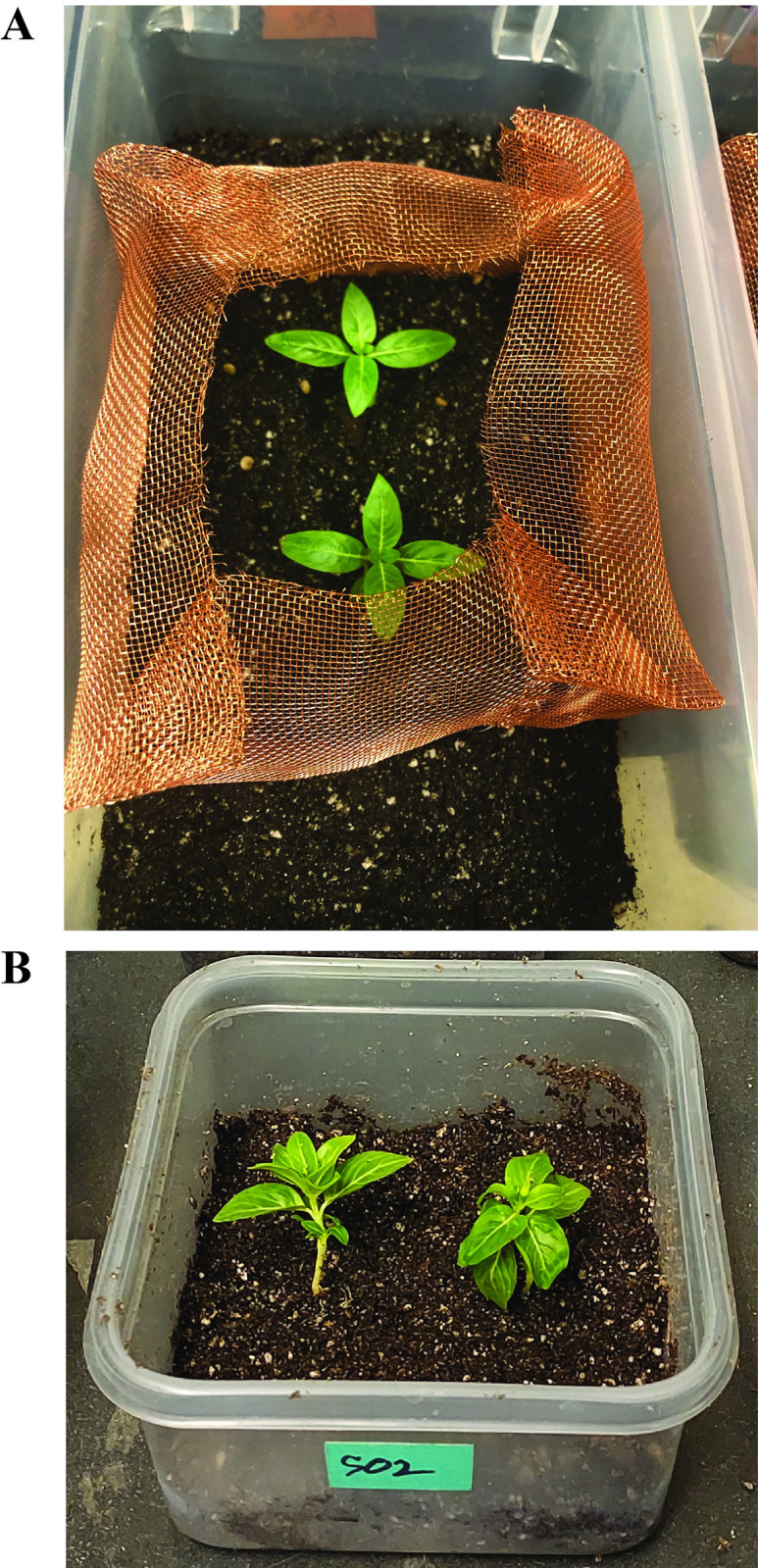
Treatment arena for determining the lethality of three US strains of *Phasmarhabditis californica* (ITD726), *P*. *papillosa* (ITD510), and *P*. *hermaphrodita* (ITD272) against (11.5-15mm/0.4–1.2 gram) *Theba pisana* at 5 times recommended dose of 150IJs/cm^2^ (A) and for the dosage dependence of *P*. *californica* lethality against (11.5-15mm/0.5–1.3 gram) *T*. *pisana* at 30 IJs/cm^2^ (Nemaslug® recommended dose) and 90 IJs/cm^2^ (B).

The test arenas used for testing *T*. *pisana* against 5 times the recommended dose (150 IJs/cm^2^) of all 3 *Phasmarhabditis* species and a recommended dose of Sluggo Plus® consisted of a container (33.5cm L x 11.5cm H x 18.5cm W) filled with 3 layers of (a) pea gravel (350mL) at the bottom, (b) a fabric barrier (Dewitt 3’ x 100’ 6 Year Weed-Barrier Landscape Fabric) that fitted the tray and (c) 600g of autoclaved soil (75% SunGro Sunshine No. 4 mix and 25% UC soil mix 3) [58]. Six hundred milliliters of deionized water was added to each arena to adjust the soil moisture. Two 6-week-old periwinkle (*Vinca minor* L.) were planted 3cm to the left and right of the arena’s center as a possible substrate for aestivation. A 16.5cm^2^ area in the middle of the arena was enclosed with a copper wire to prevent snail escape and to limit the area of application (Fig 2A). Additionally, the lid with multiple holes was placed on top of the arena. *T*. *pisana* used in this study ranged from 11.5-15mm wide and 0.4–1.2 grams.

### Dose dependence assay on *Theba pisana* exposed to Phasmarhabditis californica

Arenas used to assay *T*. *pisana* against the recommended Nemaslug ® dose (30 IJs/cm^2^) and 3 times (90 IJs/cm^2^) this recommended dose, as well as the recommended dose of Sluggo Plus® consisted of a 1436.5cm^3^ (13cm x 13cm x 8.5cm) container filled with 100g of the same autoclaved soil described above [58]. One hundred milliliters of deionized water was added to each arena to adjust the soil moisture. Two 1 month old periwinkle (*Vinca minor* L.) were planted about 4cm from the edge (Fig 2B). A lid with multiple holes was placed on top of the arena to prevent snail escape. This smaller arena was used due to limited space within the laboratory temperature-controlled incubator. The snails used in this study were within the same size range as previously described, however their weights ranged from 0.5–1.3 gram.

### Nematode preparation

The IJs used for inoculation were prepared using a modified white trap method [59] using frozen *Ambigolimax valentianus* Ferussac, 1822 inoculated with mixed stages of each *Phasmarhabditis* species. *A*. *valentianus* within the white traps were inoculated with xenic cultures of *P*. *californica* (ITD726), *P*. *hermaphrodita* (ITD272), and *P*. *papillosa* (ITD510). Infective juveniles were the only stage of nematode used throughout all experiments. All IJs were collected from the modified white traps and were stored in tissue culture flasks. IJs were quantified within a tissue culture flask by counting their number in a 10μL drop of water five times and calculating the average IJs/10μL and using this to determine the required volume/arena. The necessary volume of IJs was pipetted into individual conical tubes, and the final volume was adjusted to 10mL using double distilled water prior to application.

The higher recommended dose of 4.88kg/m^2^ of iron phosphate (Sluggo Plus®, active ingredients (a.i.) are: 0.97% iron phosphate and 0.07% Spinosad (a mixture of spinosyn A and spinosyn D)) was used as the control molluscicide. A no-nematode, snail-only treatment was also added for comparison. Sluggo Plus® was chosen for use instead of the more popular metaldehyde baits because of its recent increase in popularity which came about due to metaldehyde bait’s known off-target effects.

### Experimental set-up

Multiple locations within San Diego County, California were previously identified to have large populations of invasive *T*. *pisana*. These locations were previously identified by the Area IPM Advisor Cheryl Wilen. The snails were collected under CDFA permit 3449 from an empty non-cultivated grassy field near a commercial lot, adjacent to multiple commercial buildings in Carlsbad, California (33.1289523, -117.2489610).

**Fig 3:**
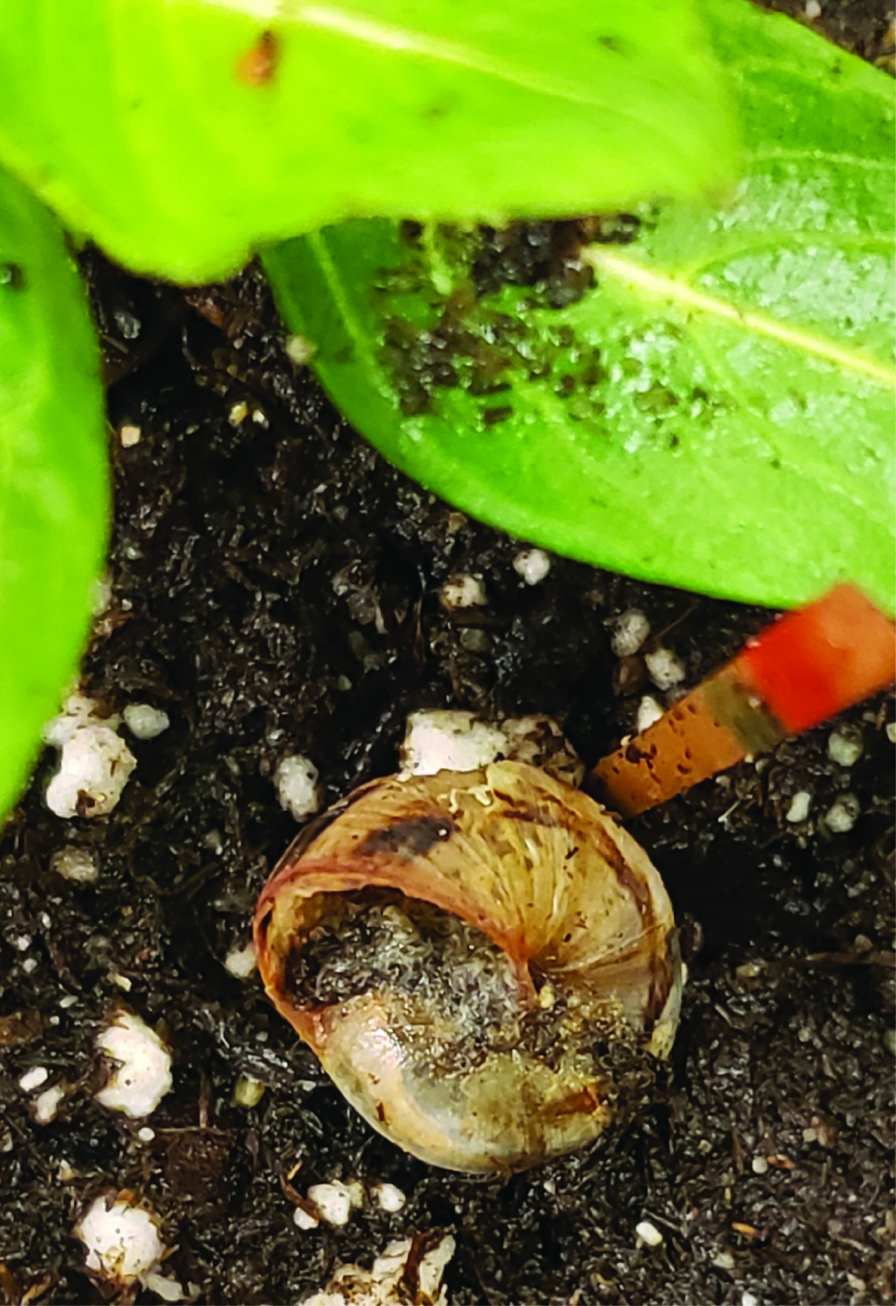
A dead adult *Theba pisana* snail with mixed stages of *Phasmarhabditis californica* (ITD726) within the shell cavity.

Ten pre-weighed snails of the mentioned weight range were divided into 3 different groups of light, medium, and heavy corresponding to 3 replicates for each trial to avoid size bias. The snails were introduced to the soil around the *V*. *minor* plants. After snail introduction, the nematode inoculum was applied evenly to the arena using an auto pipettor. The number of dead snails were recorded daily for 2 weeks. Dead snails were identified by placing a toothpick next to a suspected dead snail. If the dead snail suspect did not move from the same toothpick location after 24 hours, and the snail did not respond to prodding with a toothpick, the snail was considered dead, and the toothpick was colored red to indicate death. Additionally, dead snails usually had withdrawn foot muscles without the presence of dried mucus (epiphragm) that is typically produced to prevent desiccation during aestivation. Also, all dead snails had the presence of mixed stage nematodes present within their shell, and throughout the body (Fig 3). All experimental trials had three replicates and were repeated thrice. The 1^st^ series of assays to compare the lethality of all 3 *Phasmarhabditis* spp. at 5-fold the recommended dose were performed inside a diurnal growth incubator with alternating temperatures of 20°C and 15°C for a 12-hour day/night cycle. The 2^nd^ series to assess the lethality of *P*. *californica* at the Nemaslug ® -recommended dose and 3 –fold this dose was performed on a benchtop in the laboratory at room temperature (~23°C). All arenas were covered with a fabric barrier (Dewitt 3’ x 100’ 6 Year Weed-Barrier Landscape Fabric) to prevent excess light exposure.

All statistical analyses were performed with GraphPad Prism 9, utilizing Mantel-Cox log-rank analyses to compare each treatment to each other.

## Results

### Lethality of *Phasmarhabditis papillosa*, *P*. *californica*, and *P*. *hermaphrodita* at 5-fold the recommended dose (150 IJs/cm^2^) against *Theba pisana*

**Fig 4:**
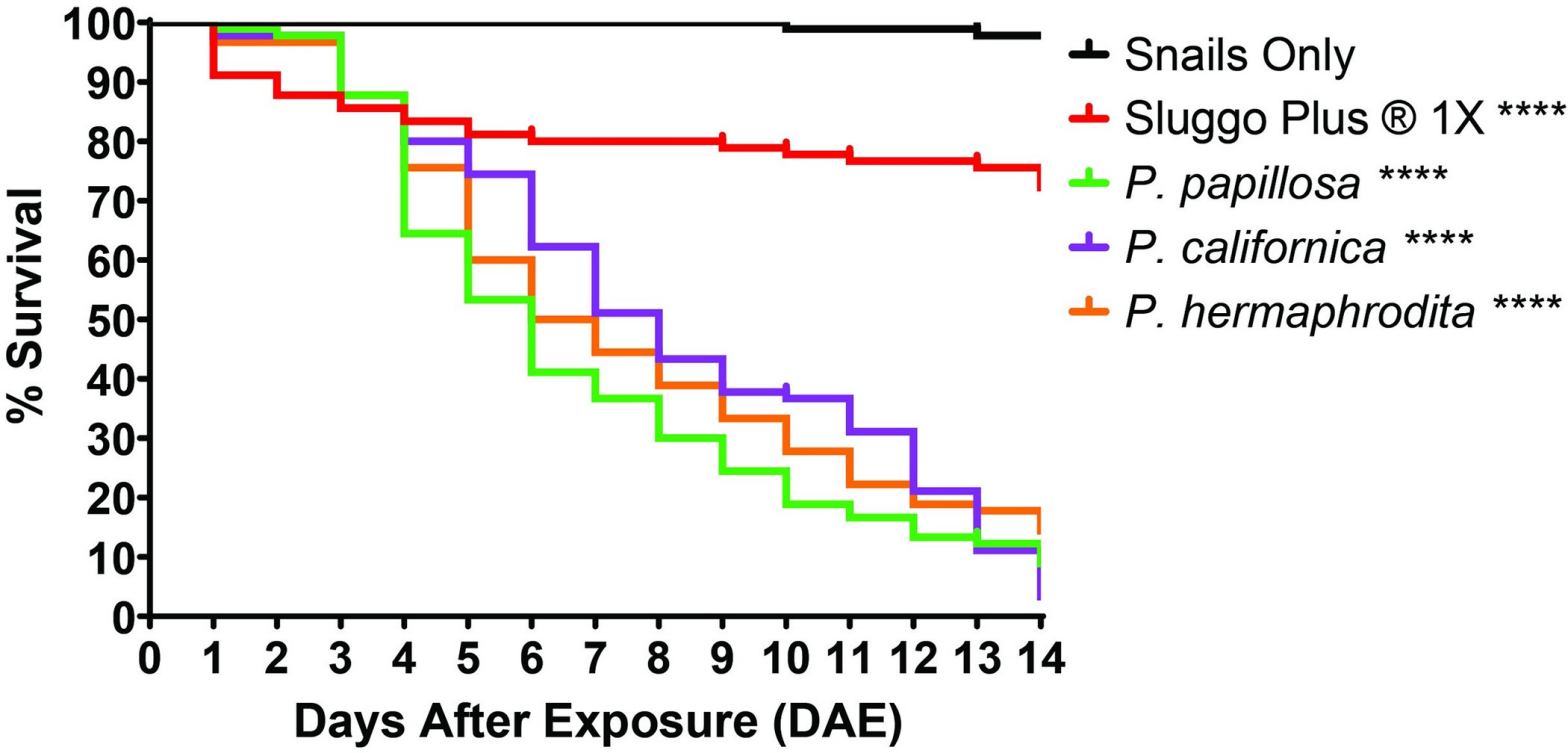
Kaplan Meier graph showing the percent survival of large adult *Theba pisana* over 14 days after exposure to 5 times the Nemaslug ®-recommended dose (150 IJs/cm^2^) of three US strains of *Phasmarhabditis californica* (ITD726), *P*. *papillosa* (ITD510), and *P*. *hermaphrodita* (ITD271), and Sluggo Plus®. The snails only control included a treatment with no application of *Phasmarhabditis*. **** indicates a p value less than 0.0001 compared to untreated control. Statistical analyses were performed by doing Mantel-Cox log rank analyses comparing each treatment to each other.

Application of *P*. *papillosa*, *P*. *californica*, and *P*. *hermaphrodita*, at 5 times the recommended dose (150 IJs/cm^2^) resulted in significant mortality after 2 weeks compared to both untreated control and the molluscicide Sluggo Plus® (p < 0.0001 for all treatments compared to control and Sluggo Plus®) (Fig 4). The three US strains of *Phasmarhabditis* spp. caused between 86–97% mortality after 14 days. There were no statistical differences between *Phasmarhabditis* treatments (p > 0.05). *P*. *californica* caused the highest mean mortality of 97% 14 days after exposure (DAE), followed by *P*. *papillosa* (91%) and *P*. *hermaphrodita* (86%) (Fig 4). Sluggo Plus® also caused significant mean mortality of 28% compared to the untreated control, 14 DAE (p < 0.0001), however it caused significantly less mortality compared to all three US *Phasmarhabditis* spp. (p > 0.05) (Fig 4).

### Lethality of *Phasmarhabditis californica* at the recommended (30 IJs/cm^2^) and 3-fold (90 IJs/cm^2^) dose against *Theba pisana*

**Fig 5:**
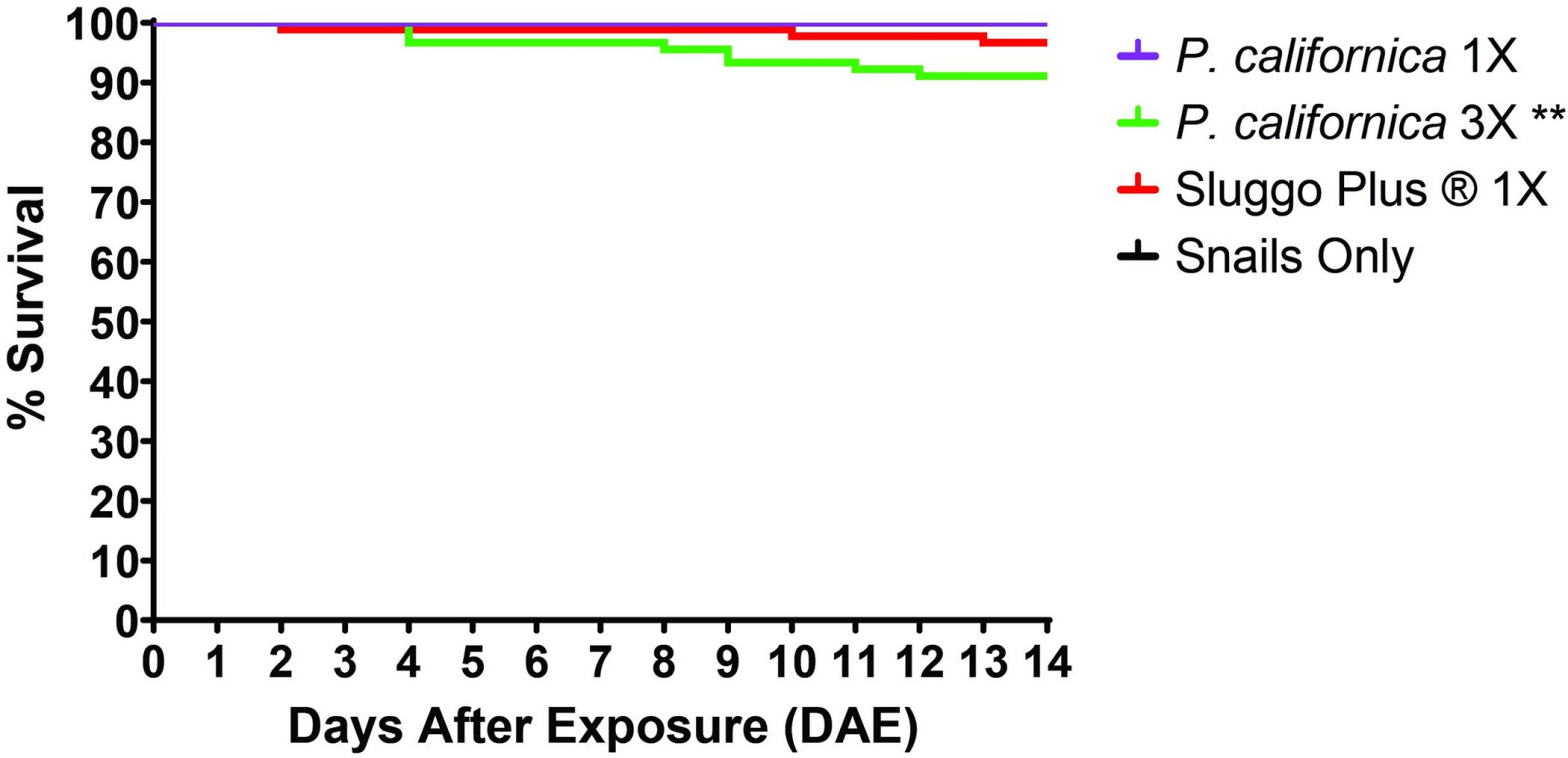
Kaplan Meier graph showing the percent survival of adult *Theba pisana* over 14 days after exposure to the Nemaslug ®-recommended dose (30 IJs/cm^2^) and three times the recommended dose (90 IJs/cm^2^) of *Phasmarhabditis californica* (ITD726) and Sluggo Plus®. ** indicates a p value less than 0.01 compared to the untreated (snails only) control. Statistical analyses were performed by doing Mantel-Cox log rank analyses comparing each treatment to each other.

Treatment of *P*. *californica* at the Nemaslug ®-recommended dose (30IJs/cm^2^) did not cause significant mean mortality (3.3%) compared to the untreated control (p > 0.9999) 14 DAE (Fig 5). However, at 3 times the recommended dose (90 IJs/cm^2^) snail mortality (8.9%) was highly significant compared to the snail only control (p < 0.0039) (Fig 5). Interestingly, treatment with Sluggo Plus® in the *P*. *californica* dosage assay did not cause significant mortality compared to the untreated control (p = 0.0815), whereas it caused significant mortality in the previous comparative assay of 3 *Phasmarhabditis* spp. (Figs 4 and 5). While the 3 times dose caused significant mortality of *T*. *pisana* compared to the snail only/untreated control, the mortality rate was low enough to disregard the dose for potential use.

## Discussion

The three US strains of *Phasmarhabditis* spp. (*P*. *californica*, *P*. *hermaphrodita*, and *P*. *papillosa*) caused mortality in both adult and juvenile *T*. *pisana*. All three caused significantly higher mortality in adult *T*. *pisana* compared to the chemical molluscicide Sluggo Plus® and the control when applied at 5 times the recommended dose of 150 IJs/cm^2^ (Fig 4). Compared to our previous findings on the efficacy of these *Phasmarhabditis* spp. to smaller-sized (2-6mm) juvenile *T*. *pisana*, it takes about 9 more days for the same level of mortality to be observed in larger adult *T*. *pisana* [60]. This result shows a size-dependent response to *Phasmarhabditis* exposure, similar to Glen *et al*. (1996) and Speiser *et al*. (2001), but adult snails were not capable of successfully fending off nematode infection. Our results agree with previous findings on naturally occurring *Phasmarhabditis* in France, where strains of *P*. *hermaphrodita* isolated from *T*. *pisana* and *Trochoidea elegans* (Gmelin, 1791) caused similar mortality rates (100% mortality 6 DAE) when applied to snails less than 6mm and snails larger than 10mm [61]. However, previous work also found that the smaller snails died at a faster rate than the larger snails [61].

Multiple studies have shown that *Phasmarhabditis’* efficacy as a biological control agent is dependent on the size or life stage of the gastropod host. Tested species of gastropod neonates have been found to be more susceptible to *Phasmarhabditis* infection and mortality, and adults have increased resistance or immunity to *Phasmarhabditis* infection [46,57,62,63]. *Phasmarhabditis* resistance also seems to be species specific. For example, *D*. *reticulatum* seems to be susceptible to multiple *Phasmarhabditis* species at various sizes throughout all life stages [44,52,64]. Multiple other slug and snail species have been tested for susceptibility to *Phasmarhabditis* as well, and there are varying mortality results for each gastropod species [41,42,46].

The lower dosage (30 IJs/cm^2^ and 90 IJs/cm^2^) assay showed that a more economically sound dose of *P*. *californica* and likely other species of *Phasmarhabditis* may not be effective against *T*. *pisana*. This is because *P*. *californica* was the most virulent of the three tested species in the 5X lethality assay and it was incapable of causing efficient mortality at lower doses (Fig 5). The best result using lower doses only caused a mortality rate of about 9% after 14 days (Fig 5). This is in congruence with previous studies done with *P*. *californica* on the brown garden snail *Cornu aspersum*. Other researchers found that *P*. *californica* was not capable of causing significant mortality to the snail 21 DAE at the recommended rate (30 IJs/cm^2^) [60]. However, *P*. *californica* has been found to kill *D*. *reticulatum* at a lower, more economically feasible dose. One study found that *P*. *californica* was able to cause significant mortality at about 45IJs/cm^2^ and 90 IJs/cm^2^ [44]. However, it took significantly longer to cause mortality in the slugs at lower doses.

The Sluggo Plus® control in the lower dose assay did not cause similar mortality to what was observed in the 5X pathogenicity assay even though the same concentration of Sluggo Plus® was provided. In the lower dose assay, Sluggo Plus caused a mortality rate of only about 3%, whereas, in the assay that used a 5X dose of US *Phasmarhabditis* strains, the same Sluggo Plus® caused a mortality rate of about 28% (Figs 4 and 5). This discrepancy could have occurred for a variety of reasons. There were differences between each of the assays, specifically the arena design. The lower dose assay was not performed in an incubator; therefore, there were no day/night-controlled temperature cycles. Instead, the arenas were consistently at room temperature which fluctuated between about 21-23C. The lower dose assay also had no copper barriers in use due to the size of the arenas. It is possible that the copper barriers in the 5X pathogenicity assay forced an increased exposure of treatment to the snails, or perhaps even decreased the snail’s health itself. It has been found that copper carbonate is toxic to some aquatic and semiaquatic snails [65–67]. Copper carbonate forms when copper is exposed to moisture. Therefore, it is possible that the snails in assays that used copper barriers were exposed to copper carbonate, which helped increase observed mortality rates. Exposure to copper carbonate could have come from direct contact of the copper barrier, or from leaching into the soil. However, experiments assessing copper carbonate’s toxicity to gastropods are performed in liquid exposure [65–67]. The toxicity of solid copper carbonate on snails has not been evaluated and therefore no immediate conclusions about the use of copper barriers can be made. However, copper hydroxide, a common occurrent in some soil fertilizers, has been shown to be toxic to some terrestrial snails, including *T*. *pisana* [68]. While copper barriers would not have led to exposure to copper hydroxide, exposure to multiple copper compounds in both solid and liquid forms should be explored in the future.

*T*. *pisana* are known to climb up tall structures or plants in the spring or summertime when temperatures begin to increase. They do this to aestivate and protect themselves from desiccation [69]. *Phasmarhabditis* nematodes are not likely to climb up tall structures or plants, as they are susceptible to desiccation. However, *T*. *pisana* often lay eggs on moist soil, and during the late spring, young snails emerge and begin to climb. This means that there is a period when the snail progeny is disproportionately located on the soil surface. Soil application of *Phasmarhabditis* as a biological control agent is therefore likely more beneficial when applied during periods of snail emergence or during time periods when snails are laying eggs. *Phasmarhabditis* could also be sprayed on plants as needed for immediate application, however the nematodes would not persist in such applications. Further research is needed to identify methods of *Phasmarhabditis* application to target *T*. *pisana*. However, our data suggest that *Phasmarhabditis* may be a feasible option for the mitigation of *T*. *pisana* due to its specificity to gastropods and safety to other non-target species.
